# Symptomatic creatine phosphokinase elevation in a Crohn's disease patient caused by upadacitinib

**DOI:** 10.1002/ccr3.8227

**Published:** 2024-01-19

**Authors:** Anna Schuitema, Suzanne I. Anjie, Agnies M. van Eeghen, Sander W. Tas, Mark Löwenberg

**Affiliations:** ^1^ Department of Gastroenterology and Hepatology Amsterdam UMC, Location Academic Medical Center, University of Amsterdam Amsterdam The Netherlands; ^2^ 's Heeren Loo Amersfoort The Netherlands; ^3^ Department of Pediatrics, Emma Center for Personalized Medicine Amsterdam UMC, Location Academic Medical Center, University of Amsterdam Amsterdam The Netherlands; ^4^ Department of Rheumatology Amsterdam UMC, Location Academic Medical Center, University of Amsterdam Amsterdam The Netherlands

**Keywords:** CPK elevation, Crohn's disease, myopathy, upadacitinib

## Abstract

We present a Crohn's disease patient receiving high dose upadacitinib treatment with elevated CPK levels and myopathy, and provide the reader with practical tips on stopping and restarting upadacitinib, emphasizing the need for adequate monitoring.

## 1 CASE PRESENTATION

Here, we present a 49‐year‐old female patient with a history of Crohn's disease (CD) and axial spondyloarthritis, who developed symptoms of myopathy 1 week after dose increase with upadacitinib from 30 to 45 mg once daily (QD). Laboratory investigation revealed an elevated creatine phosphokinase (CPK) serum concentration and a low hemoglobin level. Symptoms resolved and CPK levels normalized 2 weeks after discontinuation of upadacitinib. Treatment with upadacitinib was restarted at a dose of 15 mg QD, which was effective and could be continued safely without side effects. Follow‐up laboratory investigations showed normal CPK levels.

## 2 INTRODUCTION

Janus kinase (JAK) inhibitors are small molecule therapeutic agents that suppress the JAK‐signal transducer and activator of transcription (STAT) signaling pathway, which is implicated in the pathogenesis of various chronic inflammatory diseases.^1^ Upadacitinib is a JAK‐1/3 inhibitor, which is registered for the treatment of rheumatoid arthritis, psoriatic arthritis, axial spondyloarthritis, atopic dermatitis and ulcerative colitis. The dose varies per disease entity and treatment phase, ranging from 15 mg to 45 mg QD.^2^ Clinical trials revealed that upadacitinib is also efficacious in moderate‐to‐severe CD.^3^, ^4^ The EMA and FDA recently approved upadacitinib as treatment for moderately to severely active CD at an induction and maintenance dose of 45 mg and 15 or 30 mg QD, respectively.^2^, ^5^ Treatment with JAK inhibitors is associated with, often asymptomatic, increased CPK levels, which suggests that this agent stimulates muscle degeneration.^6^, ^7^ Stimulation of myoblast differentiation, due to suppressed inflammatory cytokine synthesis, is an alternative mechanism that has been proposed.^8^ Myoblast differentiation, in turn, can result in elevated CPK levels, but it is thought that this rarely leads to clinical symptoms. To our knowledge, we here present the first case of upadacitinib‐induced symptomatic CPK elevation in routine care in a CD patient.

## 3 CLINICAL CASE PRESENTATION

A 49 year‐old female patient with a medical history of axial spondyloarthritis and ileocolonic CD failed prior treatment for CD with azathioprine, mercaptopurine, methotrexate, infliximab, adalimumab, certolizumab, ustekinumab and vedolizumab. In 2006, she underwent a laparoscopic ileocecal resection due to a perforation of the terminal ileum. In 2016, a colonoscopy revealed recurrence of CD in the neo‐terminal ileum (modified Rutgeerts score: i3), after which combination treatment was started with vedolizumab and infliximab. In 2019, the vedolizumab infusion interval was shortened from every 8 to every 6 weeks, combined with a course of prednisone due to endoscopically confirmed active CD in the neo‐terminal ileum. In 2020, patient underwent a re‐resection of the neo‐terminal ileum, followed by maintenance treatment with infliximab and methotrexate. A follow‐up colonoscopy 6 months after surgery showed recurrence of CD in the neo‐terminal ileum (modified Rutgeerts score: i2b). In October 2022, treatment with upadacitinib was started at a dose of 15 mg QD by the rheumatologist according to the label. A favorable clinical response of axial spondyloarthritis‐related complaints was seen, but CD‐related symptoms persisted. The dose was increased to 30 mg QD, which was continued for a few months. In May 2023, the patient increased the upadacitinib dose to 45 mg QD on her own initiative because of progressive CD‐related complaints. After 1 week, she presented herself at our out‐patient clinic with muscle stiffness and cramps of the proximal legs, fitting with symptoms of myopathy. Laboratory investigation revealed an elevated CPK serum concentration (271 U/L), a mildly elevated alanine transaminase (ALT) level (68 U/L), and a slightly decreased hemoglobin count (7.1 mmoL/L). Leukocyte and thrombocyte counts, electrolytes (including magnesium and potassium) and fecal calprotectin levels were normal. Under the suspicion of upadacitinib‐induced symptomatic myopathy, treatment with upadacitinib was discontinued. Within 2 weeks, the CPK serum concentration normalized, the ALT level improved and symptoms of myalgia disappeared. Treatment with upadacitinib was restarted at the lowest effective dose of 15 mg QD. Up till now, the patient is in clinical and biochemical remission tolerating 15 mg QD upadacitinib, requiring no changes in medical treatment.

## 4 DISCUSSION AND TAKE HOME MESSAGE

Asymptomatic CPK elevations have been reported in atopic dermatitis, rheumatoid arthritis, psoriatic arthritis, and ankylosing spondylitis patients receiving treatment with upadacitinib and other JAK inhibitors, but the exact underlying mechanisms remain to be defined.^7^ There is a clear dose response association with JAK inhibitors: that is, higher doses are more efficacious, but on the other hand are associated with increased toxicity.^3^, ^7^ Hence, treatment effects of JAK inhibitors should be balanced against potential side effects. CPK elevation has been reported in up to 10% of patients receiving treatment with JAK inhibitors and this is mainly observed in patients receiving higher (induction) doses.^2^ However, this infrequently results in clinical symptoms and CPK elevation is rarely an indication to discontinue treatment with JAK inhibitors. In rheumatoid arthritis, two cases of symptomatic CPK elevation in patients receiving the JAK inhibitor baricitinib have been described.^9^ In these patients, myalgia symptoms resolved and CPK levels normalized quickly upon treatment cessation, similar to our case.

Upadacitinib has recently been approved as the first JAK inhibitor for CD based on the outcomes of a phase III randomized controlled trial.^4^ Especially patients receiving treatment with high(er) upadacitinib doses are at an increased risk of developing side effects, including myopathy complaints accompanied by elevated CPK levels. We here present the first patient with axial spondyloarthritis and CD in routine care with a symptomatic CPK elevation 1 week after upadacitinib dose escalation to 45 mg QD. Following discontinuation of upadacitinib treatment, clinical symptoms rapidly disappeared and CPK levels normalized. In case of upadacitinib‐induced myopathy, a drug holiday is indicated, followed by restarting upadacitinib at the lowest effective dose (15 mg QD). Disease flares during maintenance treatment can be managed with a dose increase to 30 or 45 mg QD, combined with adequate monitoring including regular laboratory check‐ups.

## AUTHOR CONTRIBUTIONS

**Anna Schuitema:** Writing – original draft; writing – review and editing. **Suzanne I. Anjie:** Supervision; writing – original draft; writing – review and editing. **Agnies M. van Eeghen:** Writing – original draft; writing – review and editing. **Sander W. Tas:** Data curation; writing – original draft; writing – review and editing. **Mark Löwenberg:** Data curation; writing – original draft; writing – review and editing.

## FUNDING INFORMATION

This case report received no specific grant from any funding.

## CONFLICT OF INTEREST STATEMENT

AS: Declares no conflicts. SA: Declares no conflicts. AE: Declares no conflicts. ST: Reports research grants from AbbVie, Eli Lilly, Arthrogen, AstraZeneca, BMS, Celgene, Galapagos, GSK, MSD, Pfizer, Roche, Sanofi‐Genzyme, all outside the submitted work. ML: reports consultancy and lecture fees from Abbvie, Bristol Myers Squibb, Eli Lilly, Galapagos, Janssen‐Cilag, Johnson & Johnson, Medtronic, Pfizer, Takeda, Tillotts. He is a central reader for Alimentiv and has received research grants from Galapagos and Pfizer, all outside the submitted work.

## CONSENT

Written informed consent was obtained from the patient to publish this report in accordance with the journal's consent policy.

## Data Availability

The data underlying this case report will not be shared publicly to respect the privacy of the patient described here.
